# Potential of multidetector computed tomography and magnetic resonance imaging in quantifying left ventricular function, perfusion and viability of chronic microinfarction

**DOI:** 10.1186/1532-429X-11-S1-O24

**Published:** 2009-01-28

**Authors:** Marcus Carlsson, David Saloner, Alastair Martin, Loi Do, Maythem Saeed

**Affiliations:** 1 Clinical Physiology, Lund University Hospital, Lund, Sweden; 2 Dep of Radiology and Biomedical Imaging, San Francisco, CA USA

**Keywords:** Magnetic Resonance Imaging, Multidetector Compute Tomography, Cine Imaging, Regional Perfusion, Perfusion Deficit

## Introduction

Microinfarction is common following coronary interventions (PCI and CABG) and acute coronary syndromes. Magnetic resonance imaging (MRI) has become the reference method for non-invasive quantification of: 1) left ventricular (LV) function using cine imaging and 2) myocardial perfusion deficits using first pass perfusion and 3) acute and chronic myocardial contiguous infarction using delayed contrast enhancement (DE) technique. Modern multidetector computed tomography (MDCT) has also been recently used in assessing the above parameters in contiguous infarction. To our knowledge both modalities have not been tested in the assessment of microinfarction caused by microembolic agents.

## Purpose

This study aimed to examine the potential of 64-slice MDCT and MRI in assessing LV function, regional perfusion and viability in microinfarction.

## Methods

**Figure 1:**
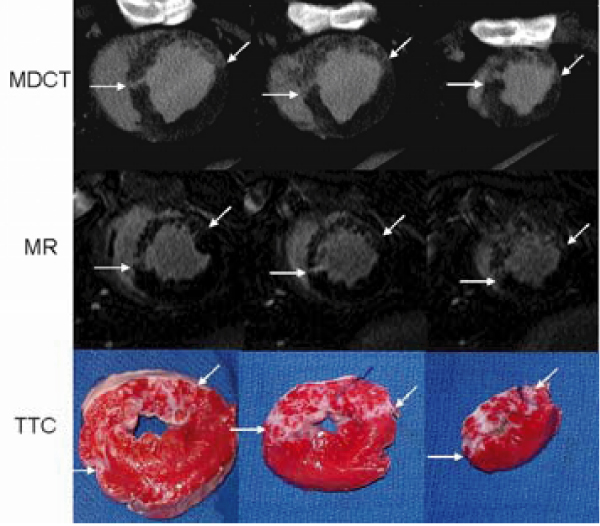
**Multi-slice MDCT (top), MR delayed enhancement images (middle) and TTC slices (bottom) from a representative animal show good correspondence between modalities in defining patchy microinfarction embedded in viable myocardium (indicated by arrows)**.

An XMR-suite was used to catheterize the LAD coronary artery under X-ray and to define the LAD-territory using first-pass MRI during intracoronary injection of 10% Gd-DOTA. The perfusion territory was selectively microembolized in six pigs using a microembolic agent (40–120 μm, 250,000 count). LV function (cine imaging), perfusion (first-pass imaging) and viability (DE imaging) were determined 7–8 weeks after microembolization using MRI followed by MDCT (5 days later). Histochemical staining (TTC) was used as golden standard for quantification of microinfarction. Figure 1.

## Results

The LAD-territory was 32.4 ± 3.8% of the LV mass. There was no significant difference between MR and MDCT measurements of systolic wall thickening. Global LV function did not differ between MRI (end diastolic volume: 92 ± 8 ml, end-systolic volume 48 ± 5 ml and ejection fraction: 47 ± 3%) and MDCT (96 ± 8 ml, 49 ± 3 ml, 49 ± 2%, respectively). Both MRI and MDCT showed a decrease in function in the microembolized territory and an increased contractility in remote myocardium. There was no difference in the quantification of systolic wall thickening (radial strain) between the methods (*P* = ns for all segments, bias 0.3 ± 21.9%). MRI, but not MDCT, detected a perfusion deficit in the microembolized territory (significant decrease in max upslope and maximum signal intensity). Microinfarction size did not differ between MDCT (6.3 ± 0.8%LV), MRI (6.6 ± 0.5%LV) or TTC (7.0 ± 0.6%LV). Bias (Bland-Altman test) for quantifying microinfarction on MDCT was -0.6 ± 1.9%LV compared to TTC and for MRI -0.4 ± 1.3%LV compared to TTC.

## Conclusion

Modern MDCT and MRI techniques have the sensitivity to: 1) visualize and quantify chronic microinfarction and 2) demonstrate regional LV dysfunction. MRI, but not MDCT, has the sensitivity for detecting small changes in regional perfusion of chronic microinfarction. Close agreements were found between MDCT and MRI in measuring regional and global LV function. The results of this study suggest that MDCT and MRI can be used for detecting the consequences of microinfarction following coronary interventions and evaluating the efficacy of new therapies and devices designed to prevent microembolization.

